# Longstanding survival without cancer progression in a patient affected by endometrial carcinoma treated primarily with leuprolide

**DOI:** 10.1054/bjoc.2001.1900

**Published:** 2001-08

**Authors:** I Noci, P Borri, G Bonfirraro, O Chieffi, A Arcangeli, A Cherubini, S Dabizzi, A M Buccoliero, M Paglierani, G L Taddei

**Affiliations:** Department of Gynaecology, Perinatal Medicine and Human Reproduction, University of Florence, viale G. B. Morgagni 85, 50134, Firenze; Department of Experimental Pathology and Oncology, University of Florence, viale G. B. Morgagni 50, 50134, Firenze; Department of Human Pathology and Oncology, University of Florence, viale G.B. Morgagni 85, Firenze, 50134, Italy

**Keywords:** endometrial cancer, LHRH analogues, leuprolide

## Abstract

We report here a case of a patient affected by endometrial cancer and treated primarily with leuprolide, the surgical approach being unfeasible due to her compromised conditions. The therapy was continued for more than 6 years, and no progression of the disease was observed. During this period, some histological and immunohistochemical evaluations of the tumour (morphology, grading, proliferation and apoptotic index, E-cadherin expression) were performed. Furthermore, the expression of m-RNA for luteinizing-hormone releasing hormone (LHRH) receptors was determined. The results showed a discrepancy between some biological parameters of the tumour and its clinical characteristics. In fact, despite features suggestive of a progression of the cancer (such as the increase of both tumour grading and proliferating capacity (MIB-1), and a fall in the reparative process (appearance of mutated p53, reduced expression of both bcl-2 and c-erb-2) being detected, neither local invasion nor metastatic lesions were clinically observed. This discrepancy might be due to the maintenance of high levels of E-cadhezin. Moreover, since this tumour was shown to express mRNA for LHRH receptors, new evidence is provided about the favourable impact of LHRH analogue treatment in patients affected by endometrial cancer. © 2001 Cancer Research Campaign http://www.bjcancer.com


British Journal of Cancer (2001) 85(3), 333–336
© 2001 Cancer Research Campaign
doi: 10.1054/ bjoc.2001.1900, available online at http://www.idealibrary.com on IDEAL®

http://www.bjcancer.com

### Longstanding survival without cancer progression in a patient affected by endometrial carcinoma treated primarily with leuprolide

**I Noci[1], P Borri[1], G Bonfirraro[1], O Chieffi[1], A Arcangeli[2], A Cherubini[2], S Dabizzi[2], AM Buccoliero[3], M Paglierani[3] and GL Taddei[3]**

[1]Department of Gynaecology, Perinatal Medicine and Human Reproduction, University of Florence, viale G. B. Morgagni 85, 50134 Firenze; [2]Department of Experimental Pathology and Oncology, University of Florence, viale G. B. Morgagni 50, 50134 Firenze; [3]Department of Human Pathology and Oncology, University of Florence, viale G.B. Morgagni 85, 50134 Firenze, Italy

**Summary** We report here a case of a patient affected by endometrial cancer and treated primarily with leuprolide, the surgical approach being unfeasible due to her compromised conditions. The therapy was continued for more than 6 years, and no progression of the disease was observed. During this period, some histological and immunohistochemical evaluations of the tumour (morphology, grading, proliferation and apoptotic index, E-cadherin expression) were performed. Furthermore, the expression of m-RNA for luteinizing-hormone releasing hormone (LHRH) receptors was determined. The results showed a discrepancy between some biological parameters of the tumour and its clinical characteristics. In fact, despite features suggestive of a progression of the cancer (such as the increase of both tumour grading and proliferating capacity (MIB-1), and a fall in the reparative process (appearance of mutated p53, reduced expression of both bcl-2 and c-erb-2) being detected, neither local invasion nor metastatic lesions were clinically observed. This discrepancy might be due to the maintenance of high levels of E-cadhezin. Moreover, since this tumour was shown to express mRNA for LHRH receptors, new evidence is provided about the favourable impact of LHRH analogue treatment in patients affected by endometrial cancer. © 2001 Cancer Research Campaign http://www.bjcancer.com

**Keywords**: endometrial cancer; LHRH analogues; leuprolide

Thus far few clinical trials have been performed with the aim of treating patients affected by endometrial cancer with luteinizing hormone-releasing hormone (LHRH) analogues. Moreover, from such studies often conflicting results have emerged: in fact, while Gallagher (Gallagher et al, 1991; Jeyarajah et al, 1996), De Vriese (De Vriese and Bonte, 1993) and Lhommé (Lhommé et al, 1999) documented clinical responses ranging from 8.7% (2 cases out of 23) to 57% (4 cases out of 7), Covens (Covens et al, 1997) and Markman (Markman et al, 1997) did not observe any response. In any case, all the trials were performed in patients affected by recurrent or metastatic endometrial cancer, while a study on the use of LHRH analogues as a primary treatment for this type of cancer is currently lacking. Moreover, no data on the LHRH receptor status was reported in the above-cited studies.

In this study, we report the case of a patient affected by endometrial cancer and treated primarily with LHRH analogue, the surgical approach being unfeasible due to her compromised health. The therapy was carried on for more than 6 years, and no progression of the disease was observed. During this period, some histological and immunohistochemical evaluations of the tumour, including morphology, grading, proliferating index and apoptotic power, were performed. Moreover, the expression of m-RNA for LHRH receptors was determined.

*Received 15 November 2000*
*Revised 14 March 2001*
*Accepted 2 May 2001*

*Correspondence to:* I Noci

The results showed a discrepancy between the biological features of the tumour (such as increase of grading and proliferating capacity, associated with a failure in the reparative process) and its clinical appearance (lack of local invasion or metastatic lesions), giving new evidence of the favourable impact of LHRH analogue treatment in patients affected by endometrial cancer.

### THE CASE

The patient BB, aged 74, para 2, with menopausal transition at 60 years, came to our care in November 1999, being previously cared for by a private gynaecologist.

She was affected by cerebellar ataxia and had had several episodes of myocardial infarction. In 1988 she underwent a curettage of the uterine cavity for metrorrhagia, with a histological diagnosis of atypical endometrial hyperplasia. Afterwards, the patient was given progesterone at high doses for 6 months and underwent a second curettage with a diagnosis of simple hyperplasia in cystic regression. In the absence of symptoms, the patient abandoned the follow up as well as the therapy. 5 years later, in February 1994, the patient had another episode of metrorrhagia: a curettage of the cavity was performed, giving rise to the diagnosis of endometrial adenocarcinoma. A surgical treatment could not be applied at that time due to the patient's severely compromised health conditions; therefore she was treated with LHRH analogues. The treatment consisted of monthly injections of 3.75 mg of leuprolide (Enantone depot 3.75 mg, Takeda Italia, Rome, Italy); the therapy was regularly administered until the time of our first observation. During the treatment, in September 1996, the patient experienced an episode of bleeding: a curettage was performed and the diagnosis of endometrial cancer confirmed.

At the time of her first admission at our clinic, the patient's weight and height were 92 kg and 168 cm, respectively, and blood pressure values 155/85 mmHg. Baseline blood parameters were in the normal range. Head and chest X-ray scans showed normal figures. The transabdominal and transvaginal ultrasound examination of the pelvis revealed a uterus bigger than expected for the menopausal period ($92 \times 45 \times 88$ cm), with thickened and non-homogeneous endometrial patterns (18 mm). The ovaries were in the normal dimensional range, and no fluid was present in the Douglas pouch. The CT scan of upper and lower abdominal quadrants confirmed the endometrial thickening and demonstrated no involvement of aortic and iliac lymph nodes and no myometrial infiltration. A curettage of the uterine cavity was performed: a specimen of the tumour was placed in buffered formaline for pathological evaluation and another, collected in sterile conditions, was frozen and stored in liquid nitrogen for molecular biology evaluations (RT-PCR assay).

Since all the endometrial specimens arising from previous curettages of the cavity had been referred to the Pathology Department of our University, the pathologist (GLT) was able to reconsider all those slides at the same time, along with the endometrial sample obtained at the time of admission to our Clinic. Moreover, an immunohistochemical study for oestrogen and progesterone receptors, proliferative index (MIB-1), p53, bcl-2, E-cadherin and c-erb-2 was performed on all endometrial samples collected so far. An informed consent from the patient for this study was obtained.

Pictures of all endometrial specimens collected from the patient, from the time of first curettage in 1988 onwards, are shown in Figure 1. All previous histopathological diagnoses were confirmed; moreover, the cancer was referred to as usual (endometroid) carcinoma. As to cancer grading, a high-grade differentiation (GI) was observed in one of the first sample (Figure 1C), whereas an intermediate-grade differentiation (G2) was found in specimens collected later, as shown in Figures 1D and 1E.

An immunohistochemical study on several consecutive sections of each endometrial specimen collected was performed, as previously reported (Noci et al, 2000). Several different primary antibodies were used: monoclonal antibody 1D5 raised against human oestrogen receptor (1:30 dilution, Bio-Genex, San Ramon, CA, USA); monoclonal antibody 1A6 raised against human progesterone receptor (1:50 dilution, Bio-Genex); monoclonal antibody MIB-1 raised against nuclear antigen Ki-67 (1:60 dilution, IMMUNOTECH, Marseilles); a murine monoclonal IgG2 antibody DO-7 raised against human p53 (1:40 dilution, DAKO A/S, Denmark), which reacts with wild-type and mutant p53 protein; a murine monoclonal (clone 124, IgG1) anti-human bcl-2 antibody (1:20 dilution, DAKO). C-erb-2 protein expression was investigated with the specific monoclonal antibody TAB 250 (1:20 dilution, ZYMED Laboratories, San Francisco, CA). For E-cadherin detection, the monoclonal antibody HECD-1 (ZYMED) was used at 1:800 dilution. Negative control experiments were performed by replacing the primary antibodies with non-immune mouse serum at an equivalent protein concentration. The positivity of oestrogen and progesterone receptors, MIB-1, p53, bcl-2, E-cadherin and c-erb-2 were evaluated by estimating the fraction of positive cells on the total number of neoplastic cells, in 10 separate fields at $40 \times 10$ HPF. The immunostaining was scored as 0(5%), 1(6–10%), 2(11–50%), 3(> 50%), according to Taskin (Taskin et al, 1997). The results of the immunohistochemical study are reported in Table 1.

Reverse transcription-polymerase chain reaction (RT-PCR) amplification studies, performed as previously reported (Noci

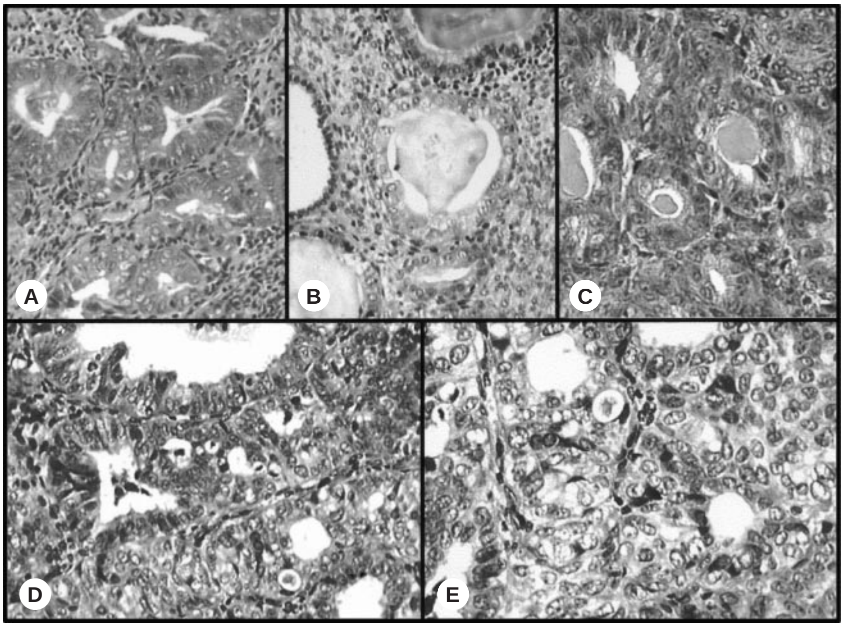

**Figure 1** Histological patterns of endometrial samples collected at different times: (**A**) hyperplastic mild hyperplasia (1988); (**B**) hyperplasia in regression, after 6 months of progesterone therapy (1989); (**C**) high-grade differentiation (G1) endometrial adenocarcinoma, referred as ordinary (usual) carcinoma, at her first diagnosis (1994); (**D**) intermediate-grade differentiation (G2) endometrial adenocarcinoma, after 2 years of therapy with LHRH analogues (1996); (**E**) intermediate-grade differentiation (G2) endometrial adenocarcinoma, during last admission, after 69 months of therapy with LHRH-analogues (1999)

 © 2001 Cancer Research Campaign

**Table 1** Immunohistochemical results in endometrial specimens collected at different times

| Date | MIB-1 | p53 | ER | | PR | | bcl-2 | | E-cadherin | c-erb-2 |
|---|---|---|---|---|---|---|---|---|---|---|
| | | | Gl | str | Gl | str | Gl | str | | |
| 1988 | 0 | 0 | 3 | 0 | 3 | 1 | 3 | 2 | 3 | 3 |
| 1989 | 0 | 0 | 3 | 3 | 2 | 2 | 0 | 2 | 3 | 3 |
| 1994 | 1 | 0 | 3 | 1 | 3 | 1 | 3 | 1 | 3 | 3 |
| 1996 | 3 | 2 | 3 | 1 | 3 | 1 | 2 | 1 | 3 | 0 |
| 1999 | 3 | 2 | 3 | 0 | 3 | 0 | 0 | 3 | 3 | 0 |

ER = oestrogen receptor; Gl = endometrial glands; PR = progesterone receptor; str = endometrial stromal cells.
All values are expressed as Taskin score (see text) as following: 0 (≤ 5%); 1 (6–10%); 2 (11–50%); 3 (>50%).

et al, 2000) were performed only on the endometrial specimen collected in our clinic, since a freshly collected sample was required. The endometrial specimen analysed was shown to be positive for LHRH receptor mRNA (not shown).

### DISCUSSION

The main comment for the case here presented arises from the behaviour of this tumour, which was retrospectively studied for more than 6 years. During this time interval, a discrepancy between the biology of the tumour and its clinical appearance was observed. In fact, as far as the biology of the tumour is concerned, there was an increase of both tumour grading (from G1 to G2) and cancer-proliferating capacity (MIB-1 from Taskin score 1 to 3), and a failure in the reparative process (appearance of mutant form of p53, and reduced expression of both bcl-2 and c-erb-2) (Table 1). On the whole, all these findings would suggest a progression of the cancer. Conversely, the clinical course of the disease, as revealed by all the investigations performed during the admission of the patient to our clinic, was not associated either with local invasion or metastatic lesions. We propose here that this discrepancy could be explained by and related to the maintenance of high levels of E-cadherin expression (Taskin score 3: see Table 1) along with time.

E-cadherins are members of a family of transmembrane glycoproteins mediating cell–cell adhesion. They are expressed by epithelial cells and are believed to play a key role in cellular organisation, tissue remodelling and in mediating extracellular signalling (Nelson et al, 1990; Magee and Buxton, 1991). It has been proposed that loss of E-cadherin expression leads to dissociation of tumour cells and promotes invasion (Shimoyama and Hirohashi, 1991; Shimoyama et al, 1992; Oda et al, 1993). Consistently, E-cadherin expression has been shown to be inversely correlated with both depth of myometrial invasion and paraaortic lymph node metastasis in endometrial cancers (Sakuragi et al, 1994; Fujimoto et al, 1998). In the case presented here, E-cadherin expression was maintained for a period of several years at very high levels (Taskin score 3); therefore, the documented lack of myometrial invasion and node metastasis might be explained by this feature of the tumour. Moreover, the aforementioned papers (Sakuragi et al, 1994; Fujimoto et al, 1998) reported that E-cadherin expression decreased with the loss of differentiation of the endometrial cancer. Conversely, in our case, the E-cadherin expression remained constant and at high levels throughout time, despite the increase in tumour grading, and proliferative index as well as the loss of reparative process. Thus, the discrepancy between biological and clinical features of the tumour here studied could be associated with the discrepancy, at the histopathological levels, between E-cadherin expression and all the other parameters investigated.

We have no clear-cut explanations for this discrepancy, but some hypotheses can be proposed: as a first hypothesis, the possibility exists of the serlendipity of this result, with the above reported feature being a peculiarity of this single case. A second explanation relies on the fact that in vitro studies reported that E-cadherin expression was significantly suppressed by oestrogens, a suppression antagonized by progesterone (Fujimoto et al, 1996). In the post-menopausal period, the ovary is no longer a source of estrogens; conversely, oestrogens derive from extraglandular conversion of androgens, mainly at the fat tissue level (Grodin et al, 1973). The BMI in our patient is 32.6, so that the patient can be classified as an obese woman. In this woman, we can easily suppose a high, chronic conversion of androgens to oestrogens at the fat tissue level. Hilum cells of the post-menopausal ovary produce testosterone and Δ4-androstenedione; the function of these cells is poorly known but is influenced, probably, by gonadotropins, and especially by LH (Yen and Jaffe, 1978). In our case, the patient had been desensitized by LHRH analogues for more than 5 years; so, the LH pituitary production and the ovarian androgen secretion had been profoundly suppressed. Therefore, it is tempting to deduce that the woman experienced a reduction in the production rate of oestrogens, which can eventually account for the lack of reduced expression of E-cadherin.

The third explanation relies on the fact that, in this case, the tumour expressed mRNA for LHRH receptors. Therefore, it is reasonable that the chronic treatment with LHRH analogues might have exerted a direct or indirect effect on E-cadherin expression at the endometrial tissue level. No current data are available at the moment supporting or refusing this hypothesis.

In conclusion, although further studies are necessary, it is the authors' opinion that this case of endometrial cancer may provide new intriguing implications on the clinical effects of LHRH analogues in the treatment of patients affected by endometrial cancer.

### ACKNOWLEDGEMENTS

This work was supported by grants from Takeda Italia Farmaceutici, s.p.a., Roma, Italy. The kind revision of the English by Dr H Witchel (University of Bristol, UK) is acknowledged.

*© 2001 Cancer Research Campaign* 

### REFERENCES

Covens A, Thomas G, Shaw P, Ackerman I, Osborne R, Lukka H, Carey M, Franssen E and Roche K (1997) A phase II study of leuprolide in advanced/recurrent endometrial cancer. *Gynecologic Oncology* **64**: 126–129

De Vriese G and Bonte J (1993) Possible role for goserelin, an LH-RH agonist in the treatment of gynaecological cancers. *Eur J Gynecol Oncol* **14**(3): 187–191

Fujimoto J, Ichigo S, Hori M, Morishita S and Tamaya T (1996) Progestins and danazol effect on cell-to-cell adhesion, and E-cadherin and α-and β-catenin mRNA expressions. *J Steroid Biochem Molec Biol* **57**: 275–282

Fujimoto J, Ichigo S, Huri M and Tamaya T (1998) Expression of E-cadherin and α- and β-catenin mRNAs in uterine endometrial cancers. *Eur J Gynecol Oncol* **19**(1): 78–81

Gallagher CJ, Oliver RTD, Oram DH, Fowler CG, Blake PR, Mantell BS, Slevin ML and Hope-Stone HF (1991) A new treatment for endometrial cancer with gonadotrophin releasing-hormone analogue. *Br J Obstet Gynaecol* **98**: 1037–1041

Grodin JM, Siiteri PK and MacDonald PC (1973) Source of estrogen production in post-menopause women. *J Clin Endocrinol Metab* **36**: 207–214

Jeyarajah AR, Gallagher CJ, Blake PR, Oram DH, Dowsett M and Oliver RTD (1996) Long-term follow-up of gonadotrophin-releasing hormone analog treatment for recurrent endometrial cancer. *Gynecologic Oncology* **63**: 47–52

Lhommé C, Vennin P, Callet N, Lesimple T, Achard JL, Chauvergne J, Luporsi E, Chinet-Charrot P, Coudert B, Couette JE, Guastalla JP, Lebrun D, Ispas S and Blumberg J (1999) A multicenter phase II study with triptorelin (sustained-release LHRH agonist) in advanced or recurrent endometrial carcinoma: a french anticancer federation study. *Gynecologic Oncology* **75**: 187–193

Magee AI and Buxton RS (1991) Transmembrane molecular assemblies regulated by the greater cadherin family. *Curr Opin Cell Biol* **3**: 854–861

Markman M, Kennedy A, Webster K, Peterson G, Kulp B and Belinson J (1997) Leuprolide in the treatment of endometrial cancer. *Gynecologic Oncology* **66**: 542

Nelson WJ, Shore EM, Wang AZ and Hammerton RW (1990) Identification of a membrane-cytoskeletal complex containing the cell adhesion molecule uvomorulin (E-cadherin), ankyrin, and fodrin in Madin-Darby canine kidney epithelial cells. *J Cell Biol* **110**: 349–357

Noci I, Coronnello M, Borri P, Borrani E, Giachi M, Chieffi O, Marchionni M, Paglierani M, Buccoliero AM, Cherubini A, Arcangeli A, Mini E and Taddei G (2000) Inhibitory effect of luteinising hormone-releasing hormone on human endometrial cancer in vitro. *Cancer Letters* **150**: 71–78

Oda T, Kanai Y, Shimoyama Y, Nagafuchi A, Tsukita Sh and Hirohashi S (1993) Cloning of the human α-catenin cDNA and its aberrant mRNA in human cancer cell line. *Biochem Biophys Res Commun* **193**: 897–904

Sakuragi N, Nishiya M, Ikeda K, Ohkouch T, Furth EE, Hareyama H, Satoh C and Fujimoto S (1994) Decreased E-Cadherin expression in endometrial carcinoma is associated with tumor dedifferentiation and deep myometrial invasion. *Gynecologicic Oncology* **53**: 183–189

Shimoyama Y and Hirohashi S (1991) Expression of E- and P-cadherin in gastric carcinomas. *Cancer Res* **51**: 2185–2192

Shimoyama Y, Nagafuchi A, Fujita S, Gotoh M, Takeichi M, Tsukita SH and Hirohashi S (1992) Cadherin dysfunction in a human cancer cell line: possible involvement of loss of α-catenin expression in reduced cell-cell adhesiveness. *Cancer Res* **52**: 5770–5774

Taskin M, Lallas TA, Barber HRB and Shevchuk MM (1997) bcl-2 and p53 in endometrial adenocarcinoma. *Mod Pathol* **10**(7): 728–734

Yen SSC and Jaffe RB. Reproductive endocrinology. WB Saunders Company Ed, Philadelphia, 1978

 *© 2001 Cancer Research Campaign*

## References

[PDF_00263] Covens A., Thomas G., Shaw P., Ackerman I., Osborne R., Lukka H., Carey M., Franssen E., Roche K. (1997). A phase II study of leuprolide in advanced/recurrent endometrial cancer.. Gynecol Oncol.

[PDF_00266] De Vriese G., Bonte J. (1993). Possible role of goserelin, an LH-RH agonist in the treatment of gynaecological cancers.. Eur J Gynaecol Oncol.

[PDF_00268] Fujimoto J., Ichigo S., Hori M., Morishita S., Tamaya T. (1996). Progestins and danazol effect on cell-to-cell adhesion, and E-cadherin and alpha- and beta-catenin mRNA expressions.. J Steroid Biochem Mol Biol.

[PDF_00271] Fujimoto J., Ichigo S., Hori M., Tamaya T. (1998). Expressions of E-cadherin and alpha- and beta-catenin mRNAs in uterine endometrial cancers.. Eur J Gynaecol Oncol.

[PDF_00274] Gallagher C. J., Oliver R. T., Oram D. H., Fowler C. G., Blake P. R., Mantell B. S., Slevin M. L., Hope-Stone H. F. (1991). A new treatment for endometrial cancer with gonadotrophin releasing-hormone analogue.. Br J Obstet Gynaecol.

[PDF_00278] Grodin J. M., Siiteri P. K., MacDonald P. C. (1973). Source of estrogen production in postmenopausal women.. J Clin Endocrinol Metab.

[PDF_00280] Jeyarajah A. R., Gallagher C. J., Blake P. R., Oram D. H., Dowsett M., Fisher C., Oliver R. T. (1996). Long-term follow-up of gonadotrophin-releasing hormone analog treatment for recurrent endometrial cancer.. Gynecol Oncol.

[PDF_00283] Lhommé C., Vennin P., Callet N., Lesimple T., Achard J. L., Chauvergne J., Luporsi E., Chinet-Charrot P., Coudert B., Couette J. E. (1999). A multicenter phase II study with triptorelin (sustained-release LHRH agonist) in advanced or recurrent endometrial carcinoma: a French anticancer federation study.. Gynecol Oncol.

[PDF_00288] Magee A. I., Buxton R. S. (1991). Transmembrane molecular assemblies regulated by the greater cadherin family.. Curr Opin Cell Biol.

[PDF_00290] Markman M., Kennedy A., Webster K., Peterson G., Kulp B., Belinson J. (1997). Leuprolide in the treatment of endometrial cancer.. Gynecol Oncol.

[PDF_00293] Nelson W. J., Shore E. M., Wang A. Z., Hammerton R. W. (1990). Identification of a membrane-cytoskeletal complex containing the cell adhesion molecule uvomorulin (E-cadherin), ankyrin, and fodrin in Madin-Darby canine kidney epithelial cells.. J Cell Biol.

[PDF_00297] Noci I., Coronnello M., Borri P., Borrani E., Giachi M., Chieffi O., Marchionni M., Paglierani M., Buccoliero A. M., Cherubini A. (2000). Inhibitory effect of luteinising hormone-releasing hormone analogues on human endometrial cancer in vitro.. Cancer Lett.

[PDF_00301] Oda T., Kanai Y., Shimoyama Y., Nagafuchi A., Tsukita S., Hirohashi S. (1993). Cloning of the human alpha-catenin cDNA and its aberrant mRNA in a human cancer cell line.. Biochem Biophys Res Commun.

[PDF_00305] Sakuragi N., Nishiya M., Ikeda K., Ohkouch T., Furth E. E., Hareyama H., Satoh C., Fujimoto S. (1994). Decreased E-cadherin expression in endometrial carcinoma is associated with tumor dedifferentiation and deep myometrial invasion.. Gynecol Oncol.

[PDF_00309] Shimoyama Y., Hirohashi S. (1991). Expression of E- and P-cadherin in gastric carcinomas.. Cancer Res.

[PDF_00311] Shimoyama Y., Nagafuchi A., Fujita S., Gotoh M., Takeichi M., Tsukita S., Hirohashi S. (1992). Cadherin dysfunction in a human cancer cell line: possible involvement of loss of alpha-catenin expression in reduced cell-cell adhesiveness.. Cancer Res.

[PDF_00315] Taskin M., Lallas T. A., Barber H. R., Shevchuk M. M. (1997). bcl-2 and p53 in endometrial adenocarcinoma.. Mod Pathol.

